# Ferroptosis hijacking by *Mycobacterium tuberculosis*

**DOI:** 10.1038/s41467-023-37149-w

**Published:** 2023-03-17

**Authors:** Boyi Gan

**Affiliations:** 1 Department of Experimental Radiation Oncology, The University of Texas MD Anderson Cancer Center, Houston, TX USA; 2 The University of Texas MD Anderson UTHealth Graduate School of Biomedical Sciences, Houston, TX USA

**Keywords:** Bacterial host response, Tuberculosis

## Abstract

A recent study from *Nature Communications* reveals that *Mycobacterium tuberculosis* can hijack epigenetic machinery in host cells and induce host cell ferroptosis, which promotes pathogen pathogenicity and spread. These findings also suggest new therapeutic strategies to treat tuberculosis.

## Mycobacterium tuberculosis and ferroptosis

Tuberculosis, an infectious disease caused by infection with *Mycobacterium tuberculosis* (Mtb) bacteria in the lung and other organs, results in more than one million deaths every year and remains a significant global health threat^1^. The development of more effective therapies to eliminate this infectious disease requires a deeper understanding of interactions between Mtb and host cells^2^. Mtb infection can cause necrotic death of host cells, facilitating the release and subsequent dissemination of Mtb to other cells. Therefore, deciphering the nature of the necrotic cell death induced by Mtb is crucial for designing novel host-directed therapies to limit Mtb dissemination.

One type of necrotic cell death studied extensively in recent years is ferroptosis, which is triggered by excessive lipid peroxidation on cellular membranes^3,4^. Harmful lipid peroxides are generated by cellular metabolic activities, but under normal conditions are kept in check by various anti-oxidant defense systems. Chief among these cellular systems is glutathione peroxidase 4 (GPX4), which uses glutathione as a co-factor to detoxify lipid peroxides and maintain cell survival^5^. Disabling this major ferroptosis defense system by suppressing GPX4 expression or activity leads to unchecked lipid peroxidation, cell membrane rupture, and ferroptotic cell death^5^. Dysregulation of ferroptosis has been implicated in a variety of diseases and pathological conditions, including cancer, neurodegenerative diseases, and ischemia/reperfusion-induced organ injury^6,7^. Interestingly, a recent study showed that Mtb infection reduced GPX4 expression and increased lipid peroxidation in host cells, and Mtb infection-induced necrotic cell death in culture and pulmonary pathology in vivo were alleviated by treatment with ferroptosis inhibitors, findings that causally link ferroptosis to Mtb-induced cell death and tissue necrosis^8^. However, the molecular mechanisms underlying this regulation have remained unclear.

## Mycobacterial modulation of host cell ferroptosis via epigenetics

In a new study in *Nature Communications*^9^, Qiang et al. set out to tackle this question by exploring the potential involvement of Mtb-encoding kinases and phosphatases in modulating host cell ferroptosis, considering the known effects of these proteins on diverse cellular processes in host cells. These analyses identified protein tyrosine phosphatase A (PtpA) as a pro-ferroptosis factor. Further experiments revealed that Mtb with *ptpA* deletion (Mtb Δ*ptpA*) was less able to decrease GPX4 expression and induce ferroptosis in host cells than wild-type Mtb, and that restoring GPX4 expression in wild-type Mtb-infected host cells partially suppressed Mtb-induced ferroptosis. These data are in line with previous findings^8^ and further establish PtpA as a key Mtb effector for suppressing GPX4 expression and promoting host cell ferroptosis.

PtpA is an Mtb secretory effector protein that not only functions as a phosphatase in the cytosol but is also capable of regulating transcription in the nucleus^10^. RNA sequencing analyses showed that Mtb infection suppressed *GPX4* expression at the mRNA level, prompting the authors to hypothesize that PtpA might suppress *GPX4* transcription in the nucleus. A series of elegant experiments performed by the authors support this hypothesis. First, Qiang et al. showed that PtpA translocated into the nucleus by interacting with GDP-bound Ran GTPase (Ran-GDP; a key factor involved in protein transport between the cytosol and the nucleus), but this interaction was independent of PtpA’s phosphatase activity; importantly, a PtpA mutant that cannot interact with Ran-GDP also lost its ability to induce ferroptosis in host cells.

In addition, a yeast-two hybrid screening identified protein arginine methyltransferase 6 (PRMT6) as a PtpA-interacting protein. PRMT6 catalyzes the asymmetric dimethylation of histone H3 at arginine 2 (H3R2me2a) and suppresses gene expression^11^. Consistent with this, PRMT6 was shown to suppress *GPX4* expression in a methyltransferase-dependent manner. Notably, the ability of PtpA to regulate *GPX4* expression or ferroptosis was lost in cells deficient in *PRMT6* or cells treated with a PRMT6 inhibitor, demonstrating that PRMT6 is required for Mtb PtpA-induced ferroptosis. Additional experiments suggested that Mtb PtpA interacted with PRMT6 and promoted PRMT6’s methyltransferase activity to add H3R2me2a marks on the *GPX4* promoter, resulting in suppression of *GPX4* transcription and induction of ferroptosis. Importantly, this interaction seems to be critical for Mtb pathogenicity and dissemination in vivo: animal experiments with Mtb infection showed that Mtb harboring a PtpA mutant incapable of interacting with PRMT6, as well as Mtb Δ*ptpA*, was less able to cause pathogenicity and dissemination in the mouse lung than wild-type Mtb.

## Conclusions and future prospects

**Fig. 1:**
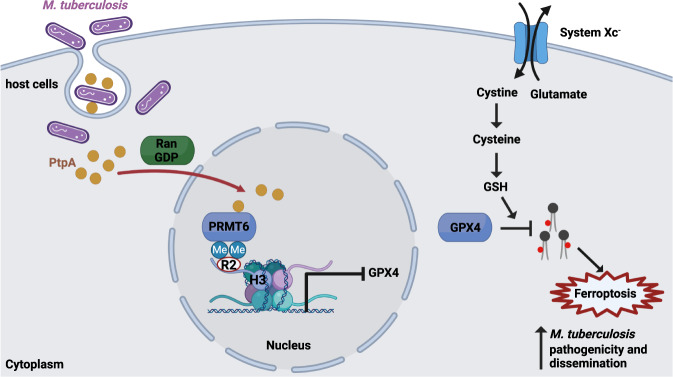
Mtb induces host cell ferroptosis to promote its pathogenicity and dissemination through its effector protein PtpA. In host cells, the system x_c_^−^ imports cystine, which is then reduced to cysteine for GSH synthesis. GPX4 utilizes GSH as a co-factor to neutralize lipid peroxides for ferroptosis suppression. Mtb-secreted effector protein PtpA enters the host cell nucleus by interacting with Ran-GTP, and promotes PRMT6-mediated H3R2me2a on the *GPX4* promoter. This results in decreased *GPX4* expression and ferroptosis induction in host cells, contributing to Mtb pathogenicity and dissemination. GSH glutathione; GPX4 glutathione peroxidase 4; Mtb *Mycobacterium tuberculosis*; PRMT6 protein arginine methyltransferase 6; PtpA protein tyrosine phosphatase A; Ran-GDP GDP-bound Ran GTPase.

Collectively, the findings by Qiang et al. unveiled a hitherto unrecognized mechanism underlying Mtb-induced ferroptosis^9^. According to their model (Fig. 1), the Mtb effector protein PtpA enters the host cell nucleus by interacting with Ran-GDP, and then interacts with PRMT6 and promotes PRMT6-mediated H3R2me2a on the *GPX4* promoter. This epigenetic regulation, which is known to be associated with gene repression, results in suppression of *GPX4* transcription and induction of ferroptosis.

These findings raise several interesting questions for future studies. On the mechanistic level, because the effect of Mtb PtpA on reducing GPX4 levels is relatively moderate, it seems less likely that such a moderate reduction in GPX4 level is sufficient to trigger strong ferroptosis in host cells. Consistent with this, the authors found that the ferroptosis inhibitor ferrostatin-1 had a more pronounced suppressive effect on Mtb-induced ferroptosis than did GPX4 restoration, suggesting that Mtb-induced ferroptosis likely involves additional mechanisms, which await further investigation in future studies. It will be particularly interesting to examine whether Mtb can directly “hijack” ferroptosis machinery in host cells, for example, through direct interactions between Mtb effector proteins and proteins involved in host-cell ferroptosis pathways or direct modulation of lipid peroxidation on host cell membranes. This study will also inspire future studies to further explore ferroptosis in other pathogenic infections. For example, another recent study showed that *P. aeruginosa* uses its effector protein pLoxA to promote lipid peroxidation and ferroptosis in human bronchial epithelial cells, which could contribute to the pathogenesis of *P. aeruginosa*-associated respiratory diseases^12^. It will be particularly interesting to explore whether there is any common underlying mechanism shared by different pathogens to manipulate host cell ferroptosis.

On the translational level, previous studies have already shown the effectiveness of vitamin E or selenium supplementation in treating patients with tuberculosis^13,14^. Vitamin E is a radical trapping antioxidant and a strong ferroptosis inhibitor, whereas selenium supplementation can boost protein synthesis of GPX4 (a selenoprotein) for ferroptosis suppression;^6^ therefore, the effect of vitamin E or selenium supplementation on treating tuberculosis can be explained, at least in part, by their roles in suppressing ferroptosis. These new findings^9^ suggest that ferroptosis inhibitors or drugs that specifically disrupt the interactions of Mtb PtpA with Ran-GDP or PRMT6 can be explored for tuberculosis treatment. More broadly, these findings^8,9,12^ together highlight the possibility of using ferroptosis inhibitors to treat diverse infectious diseases.
